# Resident training does not influence the complication risk in total knee and hip arthroplasty

**DOI:** 10.1080/17453674.2021.1979296

**Published:** 2021-10-04

**Authors:** Daphne M Bron, Nienke Wolterbeek, Rudolf W Poolman, Diederik H R Kempen, Diyar Delawi

**Affiliations:** a Department of Orthopedic Surgery, St. Antonius Hospital, Nieuwegein;;; b Department of Orthopedic Surgery, JointResearch OLVG, Amsterdam;;; c Department of Orthopedic Surgery, LUMC, Leiden, The Netherlands

## Abstract

Background and purpose — Gaining experience in the surgery room during residency is an important part of learning the skills needed to perform arthroplasties. However, in practice, patients are often not fully comfortable with trainee involvement in their own surgery. Therefore, we investigated complications, revision rates, mortality, and operative time of orthopedic surgeons and residents as primary surgeon performing total knee arthroplasties (TKAs) or total hip arthroplasties (THAs).

Patients and methods — In this multi-center retrospective cohort study, 3,098 TKAs and 4,027 THAs performed between 2007 and 2013 were analyzed. Complications, revisions, mortality, and operative time were compared for patients operated on by the orthopedic surgeon or a resident as primary surgeon. An additional analysis was performed to determine whether the complication risk was affected by the postgraduate year of the resident.

Results — Orthopedic complication rates were similar (TKA: orthopedic surgeon: 10%, resident: 11%; THA: 9% and 8%), revision rates (TKA: 3% and 2%, THA: 3% and 2%), or mortality rates (TKA: 0.1% and 0.3%, THA: 0.2% and 0.3%). For both procedures a higher non-orthopedic complication rate was found in the resident group (TKA: 8% and 10%; p = 0.03, THA: 8% and 10%; p = 0.01) and a slightly longer operative time (TKA: mean difference 9.0 minutes (8%); THA: 11.3 minutes (11%)).

Interpretation — Complications, revisions, and mortality were similar in TKAs or THAs performed by the resident as primary surgeon compared with surgeries performed by an orthopedic surgeon. This data can be used in teaching hospitals and may help to reassure patients.

In teaching hospitals, surgeries are performed by a surgeon as primary surgeon or by a resident under direct supervision of a surgeon. Gaining experience in the surgical room during orthopedic residency is an important part of learning the skills needed to perform total hip (THA) and total knee arthroplasty (TKA). However, in practice, patients are often not fully comfortable with trainee involvement in their own surgery (Nahhas et al. 2019).

A number of studies have been performed to evaluate the impact of resident involvement on complications following orthopedic surgical procedures (Schoenfeld et al. 2013, Edelstein et al. 2014, Haughom et al. 2014a, 2014b, Pugely et al. 2014, Cvetanovich et al. 2015, Bao et al. 2018). Most of these studies have used the same retrospective American National Surgical Quality Improvement Program database that collected data only up to 30 days postoperatively. Therefore, no implant-related complications such as dislocations or revisions after this time period were captured. Furthermore, the role of the resident during the procedure is missing in up to 79% of the surgeries (Edelstein et al. 2014, Haughom et al. 2014b, Cvetanovich et al. 2015, Neuwirth et al. 2018). This limits the level of evidence of the studies using this database, therefore conclusions are not reliable as to whether resident involvement in orthopedic surgical procedures is a risk factor for complications. Apart from these database studies, other studies on the effect of resident involvement were limited by small sample size or short-term follow-up. Therefore, we investigated complications, revision rates, mortality, and operation time of operations performed by an orthopedic surgeon or an orthopedic resident as primary surgeon in a large patient cohort validated with the national arthroplasty registry with over 95% completeness. We hypothesized that no clinically relevant difference in complications, revision rates, mortality, and operation time between orthopedic surgeon and orthopedic residents would be found.

## Patients and methods

This multi-center retrospective cohort study was conducted at 2 highly experienced teaching hospitals in the Netherlands. All patients who received a primary THA or TKA, in the orthopedic departments between January 2007 and December 2013, were included. There were no exclusion criteria.

All individual patient records were reviewed and used to extract data. Recorded outcomes were complications and revisions during the complete follow-up and mortality within the first 90 days postoperatively. Complications were scored as either surgical site or systemic and were divided into subgroups for different kinds of complications. A patient with more than 1 complication could be scored in more than 1 subgroup.

Collected risk factors were information on the surgeon (resident, postgraduate years of resident), duration of the surgery, age, sex, BMI, diagnosis, operation side, type of anesthesia, diabetes mellitus (DM), smoking history, and ASA score. The duration of the surgery was defined as the time between first incision until closure of the wound. All residents already had 2 years of mandatory pre-training in the general surgery department before they started with their orthopedic training, which took another 4 years. The postgraduate year (level of experience) of the resident was defined by the years in orthopedic residency so far, to determine whether an increase in experience of a resident would change the complication rates. To monitor resident training, the Entrustable Professional Activity concept is used, which allows surgeons to make competency-based decisions on the level of supervision required by residents. Four levels are used which are: assisting, operating under strict supervision, operating under limited supervision, and operating independently. Operating independently in arthroplasty is only allowed for final-year residents. Data was crosschecked with the complication registries of the departments and with the data in the Dutch Arthroplasty Register. This register has over 95% completeness (van Steenbergen et al. 2015).

### Statistics

All statistical analyses were performed with SPSS (IBM SPSS statistics, version 24; IBM Corp, Armonk, NY, USA). Unpaired 2-tailed t-tests were used to compare continue baseline data. Categorical data were compared by chi-square tests, or if not allowed Fisher’s exact tests. Binary logistic regression models for surgical site and systemic complications were created using the variables age, sex, BMI, diabetes mellitus, ASA score, primary surgeon, smoking, type of anesthesia, and operation time. The same variables were used for the THA and TKA models, except for diagnosis, which was included only in the THA model.

To determine whether experience level affects the complication risk within the resident group all resident cases were subdivided into 4 groups of increasing resident experience: first, second, third, and fourth postgraduate year. For both surgical site and systemic complications, these experience levels were compared using a chi-square test. Additionally, logistic regression analyses were done where every year in residency (risk factor) was compared with the complication rates of the orthopedic surgeon. All reported p-values were 2-tailed and for each analysis, p < 0.05 was considered significant. Odds ratios (OR) and 95% confidence intervals (CI) are reported.

### Ethics, funding, data sharing, and potential conflict of interest

The study was conducted in accordance with the Helsinki Declaration and was approved by the Institutional Review Boards of both hospitals (trialregister.nl: NL8672). Individual consent was not required. No grants were received for this study. The data that supports the findings of this study is available from the corresponding author on reasonable request. The authors declare no competing interests.

## Results

**Figure 1.**
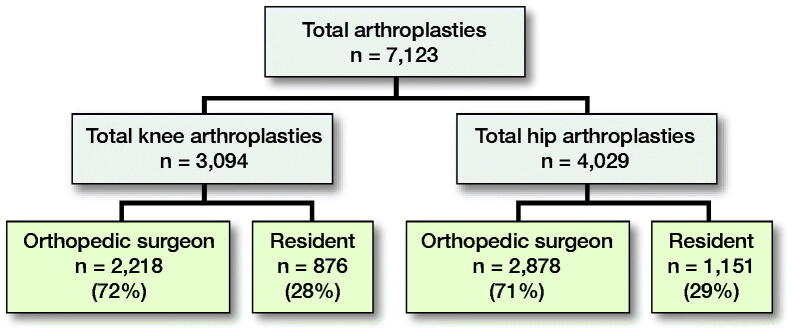
Flowchart of the number of total arthroplasties in each group.

7123 total joint arthroplasties (TJA) (3,094 TKAs and 4,029 THAs) were included with a mean follow-up time of 97 months (52–136). In the TKA group 72% of patients were operated on by the orthopedic surgeon as primary surgeon and 28% by the orthopedic resident under supervision of the orthopedic surgeon. In the THA group 71% of patients were operated by the orthopedic surgeon and 29% by the resident (Figure 1). All THAs were performed using a posterolateral approach or straight lateral (Hardinge) approach. The fixation technique was uncemented, cemented, or hybrid. For the TKAs a medial parapatellar approach was used and all TKAs were cemented. In all patients, a low-vacuum wound drain was placed and removed after 24 hours.

### Total knee arthroplasty

There were no relevant differences in age, operation side, diagnosis, DM, smoking and ASA score between groups (Table 1). There was a statistically significant difference in BMI; patients in the resident group had a slightly higher BMI (30) than patients in the orthopedic surgeon group (29). In the residents’ group 72% of the surgeries were performed under regional anesthesia versus 64% in the orthopedic surgeon group (p < 0.001). The duration of surgery was longer in the resident group (118 [SD 27] minutes) compared with the orthopedic surgeons (109 [SD27] minutes) with a mean difference of 9 minutes between groups (p < 0.001).

**Table 1.** Baseline characteristics of the patients included. For continuous data the mean and standard deviation (SD) are given, absolute and relative frequencies (%) are given for categorical data

	Total knee arthroplasty (n = 3,094)			Total hip arthroplasty (n = 4,029)		
	Orthopedic surgeon (n = 2,218)	Resident (n = 876)	p-value	Orthopedic surgeon (n = 2,878)	Resident (n = 1,151)	p-value
Age (SD)	68 (10)	69 (9)	0.2	66 (11)	71 (9)	< 0.001
BMI (SD)	29 (5)	30 (5)	0.002	27 (5)	27 (5)	0.8
Sex			0.001			< 0.001
Male	693 (31)	222 (25)		1062 (37)	347 (30)	
Female	1,522 (69)	650 (75)		1,814 (63)	804 (70)	
Operation side			0.4			0.3
Right	1,162 (53)	446 (51)		1,587 (55)	615 (53)	
Left	1,052 (48)	430 (49)		1,286 (45)	536 (47)	
Diagnosis			0.1			0.04
Osteoarthritis	2,172 (99)	866 (99)		2,551 (89)	1,049 (92)	
Other	34 (2)	6 (1)		305 (11)	98 (9)	
Type of fixation						< 0.001
Cemented	2,216 (100)	875 (100)	–	1,275 (45)	775 (67)	
Uncemented	–	–		872 (31)	201 (18)	
Hybrid	–	–		404 (14)	171 (15)	
Resurfacing	–	–		277 (10)	2 (0)	
Diabetes mellitus			0.2			0.1
No	1,638 (84)	667 (82)		2,266 (91)	953 (89)	
Yes	310 (16)	147 (18)		225 (9)	118 (11)	
Smoking			0.9			0.9
No	1,657 (87)	704 (88)		1,994 (82)	863 (82)	
Yes	243 (13)	101 (13)		439 (18)	187 (18)	
Anesthesia			< 0.001			0.3
General	774 (36)	237 (28)		1949 (70)	753 (68)	
Regional	1,356 (64)	611 (72)		851 (30)	358 (32)	
ASA score			0.05			< 0.001
I	406 (21)	139 (17)		729 (29)	217 (20)	
II	1,307 (67)	560 (69)		1,480 (60)	703 (66)	
III–IV	236 (12)	114 (14)		269 (11)	152 (14)	

#### Complications, revisions, and mortality

10% of the orthopedic surgeon group had at least 1 surgical site complication compared with 11% of the resident group (Table 2). Revision rates in both groups were 3%. There were 0.9% (peri-prosthetic) fractures in the resident group versus 0.2% in the surgeon group. There was a significant difference in systemic complications with 8% in the orthopedic surgeon group versus 10% in the resident group (p = 0.03). The difference in systemic complications was mainly seen in the number of urological complications, which consists of urinary retention and urinary tract infection. A urological complication was noted in respectively 3% versus 5% (p = 0.03). In all other subgroups, no differences were found. In the resident group, 0.2% of pneumonias were recorded versus 0.5% in the surgeon group. In the orthopedic surgeon group, 2 patients died within 90 days after surgery (1 patient with sudden death of unknown cause and 1 due to a brain stem cerebrovascular accident). In the resident group, in total, 3 patients died within 90 days after surgery (2 patients with sudden death of unknown cause and 1 from intestinal ischemia due to sepsis as a result of an infected total knee arthroplasty).

**Table 2.** Complication rates (%) and Odds ratio with 95% confidence interval (CI) of orthopedic surgeons and residents for total knee arthroplasty (N = 3,094)

Complications	Orthopedic surgeon (n = 2,218)	Resident (n = 876)	Odds-ratio (CI)
Surgical site	222 (10)	99 (11)	1.1 (0.9–1.5)
Deep infection	45 (2)	13 (2)	0.7 (0.4–1.4)
Nerve palsy	12 (0.5)	5 (0.6)	1.1 (0.4–3.0)
Intraoperative	10 (0.5)	5 (0.6)	1.3 (0.4–3.7)
Reoperation	182 (8)	83 (10)	1.2 (0.9–1.5)
Other	140 (6)	69 (8)	1.3 (0.9–1.7)
Revision	71 (3)	29 (3)	1.0 (0.7–1.6)
Systemic	175 (8)	91 (10)	1.4 (1.0–1.8)
Delirium	28 (1)	19 (2)	1.7 (1.0–3.1)
DVT or PE	15 (0.7)	7 (0.8)	1.2 (0.5–2.9)
Pulmonary	11 (0.5)	2 (0.2)	0.5 (0.1–2.1)
Urological	67 (3)	42 (5)	1.6 (1.1–2.4)
Cardiac	27 (1)	16 (2)	1.5 (0.8–2.8)
Gastrointestinal tract	9 (0.4)	3 (0.3)	0.8 (0.2–3.1)
Cerebrovascular	10 (0.5)	1 (0.1)	0.3 (0.0–2.0)
Other	36 (2)	16 (2)	1.1 (0.6–2.0)
Death within 90-days	2 (0.1)	3 (0.3)	3.8 (0.6–23)

DVT or PE: deep vein thrombosis or pulmonary embolism.

#### Independent risk factors for complications

Multivariate logistic regression analyses were performed to identify potential bias due to independent risk factors for both surgical site and systemic complications (Tables 3 and 4, see Supplementary data). Similar risks of complications were found in cases where the resident was the first surgeon. For surgical site complications, a higher age was associated with a reduction in the likelihood of exhibiting a complication (OR 1.0 for every year increase in age, p < 0.001) while for systemic complications increasing age predicted a higher complication rate (OR 1.1 for every year increase in age, p < 0.001). In addition, for surgical site complications ASA score was found to be an independent risk factor, where a higher ASA score predicted a higher complication rate (Table 3, see Supplementary data). Additional independent risk factors for systemic complications included sex: females had a lower complication rate. Diabetes and ASA score III/IV predicted a higher complication rate (Table 4, see Supplementary data).

**Table 5.** Complication rates (%) and Odds ratio with 95% confidence interval (CI) of orthopedic surgeons and residents for total hip arthroplasty (N = 4,029)

Complications	Orthopedic surgeon (n = 2,878)	Resident (n = 1,151)	Odds-ratio (CI)
Surgical site	248 (9)	91 (8)	0.9 (0.7–1.2)
Deep Infection	50 (2)	22 (2)	1.1 (0.7–1.8)
Dislocation	68 (2)	27 (2)	1.0 (0.6–1.6)
Nerve palsy	25 (0.9)	8 (0.7)	0.8 (0.4–1.8)
Intraoperative	29 (1)	10 (0.9)	0.9 (0.4–1.8)
Reoperation	151 (5)	47 (4)	0.8 (0.6–1.1)
Other	77 (3)	26 (2)	0.8 (0.5–1.3)
Revision	69 (2)	17 (2)	0.6 (0.4–1.0)
Systemic	216 (8)	116 (10)	1.4 (1.1–1.8)
Delirium	50 (2)	22 (2)	1.1 (0.7–1.8)
DVT or PE	12 (0.4)	8 (0.7)	1.7 (0.7–4.1)
Pulmonary	23 (0.8)	12 (1)	1.3 (0.6–2.6)
Urological	82 (3)	48 (4)	1.5 (1.0–2.1)
Cardiac	41 (1)	19 (2)	1.2 (0.7–2.0)
Gastrointestinal tract	12 (0.4)	7 (0.6)	1.5 (0.6–3.7)
Cerebrovascular	6 (0.2)	4 (0.3)	1.7 (0.5–5.9)
Other	33 (1)	21 (2)	1.6 (0.9–2.8)
Death within 90-days	6 (0.2)	3 (0.3)	1.3 (0.3–5.0)

DVT or PE: deep vein thrombosis or pulmonary embolism.

**Table 8.** Overall surgical site and systemic complication rates for total arthroplasty in the resident group split up for years in training compared to the surgeries performed by the orthopedic surgeon (N = 5,099)

Experience level	n	B (SE) ^a^	OR (CI) ^b^
Surgical site complication rate ^c^			
Year 1	330	–0.05 (0.2)	1.0 (0.6–1.4)
Year 2	482	–0.12 (0.2)	0.9 (0.6–1.3)
Year 3	352	0.12 (0.2)	1.1 (0.8–1.6)
Year 4	859	0.08 (0.1)	1.1 (0.9–1.4)
Systemic complication rate ^d^			
Year 1	330	0.32 (0.2)	1.4 (1.0–2.0)
Year 2	482	0.14 (0.2)	1.2 (0.8–1.6)
Year 3	352	0.12 (0.2)	1.1 (0.8–1.7)
Year 4	859	0.47 (0.1)	1.6 (1.3–2.0)

a B (SE): unstandardized regression weight with standard error.

b OR (CI): odds ratio with 95% confidence interval

c R^2^ < 0.001 (Nagelkerke). Model χ^2^(4) = 1.4, p = 0.8.

d R^2^ = 0.005 (Nagelkerke). Model χ^2^(4) = 16.5, p = 0.002.

### Total hip arthroplasty

#### Complications, revisions, and mortality

In the orthopedic surgeon group, 9% suffered at least 1 surgical site complication and in the resident group 8% (Table 5). There were 0.9% (peri-prosthetic) fractures in the resident group versus 0.4% in the surgeon group. No statistically significant differences were found when all surgical site complications were divided into subgroups. However, there were fewer systemic complications in the orthopedic surgeon group (8%) compared with the resident group (10%). Urinary tract complications (urinary retention and urinary tract infection) were more common in the resident group. In the resident group, 1% of pneumonias were recorded versus 0.6% in the surgeon group. 6 patients died in the first 90 days after surgery in the orthopedic surgeon group (1 patient with a rectus hematoma with abdominal compartment syndrome and renal failure; 1 sepsis possibly due to endocarditis; 1 out-of-hospital death of unknown cause; 1 herniation syndrome due to undiagnosed brain tumor; 1 major bleeding from severe liver cirrhosis and bleeding disorder; and 1 myocardial infarction by hemoglobin decrease in a patient with a bleeding disorder and liver cirrhosis). 3 patients died in the resident group (1 patient with massive abdominal bleeding; 1 myocardial infarction; and 1 sepsis due to early THA infection). This difference in mortality rate was not significantly different (p = 0.7).

#### Risk factors

There were no relevant differences in BMI, DM, smoking, or type of anesthesia (Table 1). There was a statistically significant difference in age, sex, diagnosis, and the type of fixation. The difference in type of fixation can be explained by the fact that the orthopedic surgeon placed more uncemented THAs and almost all resurfacing prostheses. A significant difference was found in the ASA score, where the resident operated on more ASA II and ASA III patients. The duration of the surgery was statistically significantly longer in the resident group (115 [28] minutes) than in the group with orthopedic surgeons (103 [28] minutes) with a mean difference of 11 minutes between groups.

#### Independent risk factors for complications

The risk of surgical site and systemic complications was similar for the residents and orthopedic surgeons (Tables 6 and 7, see Supplementary data). For surgical site complications a longer operative time was found as an independent risk factor (OR 1.0 for each minute’s increase in operative time) and ASA score III–IV seemed to be a predictive factor, although significance was not achieved (OR 1.6, p = 0.06). For systemic complications independent risk factors included higher age, smoking, and a higher ASA score. In addition, male sex was an independent risk factor as women had a lower complication rate (OR 0.7).

### Experience level of the resident

**Figure 2.**
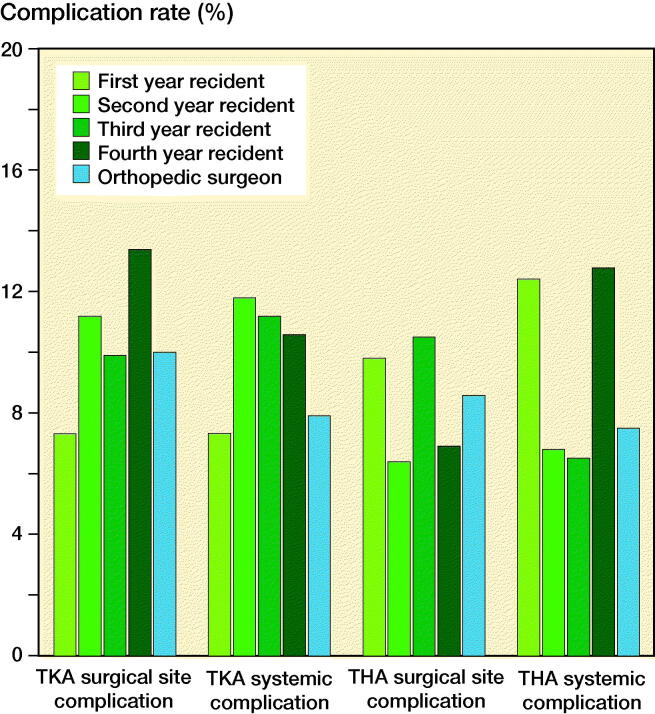
Complication rates after total knee arthroplasty (TKA) and total hip arthroplasty (THA), stratified by experience level of the resident compared with orthopedic surgeons.

The TKA and THA group were analyzed together to maximize the group sizes. Odds ratios were calculated for all postgraduate years compared with the orthopedic surgeon group. The logistic regression model for surgical site complications was not statistically significant (Table 8). For systemic complications, patients operated on by residents who were 4 years in training were 1.6 times more likely to exhibit a systemic complication compared with patients operated on by the orthopedic surgeon. For all other postgraduate years, no significant odds ratios were found. This model was statistically significant but explained only 0.5% of the variance in systemic complications (Table 8). No clinical differences in, respectively, surgical site and systemic complication rates were found when stratified by years in training (Figure 2).

## Discussion

Similar surgical site complications, revision rates, and mortality were found between surgeries performed by the orthopedic surgeon or resident as primary surgeon. However, more systemic complications were noted in the resident group for both THAs and TKAs. This seems to be caused by a larger number of urological complications. Residents operated on more patients with a higher ASA score and a higher ASA score was found to be an independent risk factor for systemic complications for both TKAs and THAs. Furthermore, THA patients in the resident group were older and a higher age in both procedures was found to be an independent risk factor for systemic complications.

We demonstrated a prolonged operative time in the resident group for both TKAs and THAs. The mean difference in operative time of 9 minutes (8%) for TKA and 11 minutes (11%) for THA were slightly less than found in previous studies, where prolonged operative times between 9% and 15% were mentioned for TKA procedures and between 17% and 22% for THA procedures when a resident was involved during surgery (Haughom et al. 2014a, 2014b). This might be explained by the residency program as Dutch orthopedic residents already have 2 years of surgical experience after fulfilling a 2-year mandatory general surgery residency program.

### Experience level of the resident

We found no relevant differences in complication rates between different levels of experience within the resident group. Only the systemic complication rate in the 4th-year resident group was higher but it explained only 0.5% of the variance in systemic complications. These results are in line with previous studies (Haughom et al. 2014a, 2014b). However, in these earlier studies, the role of the resident during surgery was not clear and the sample size was not reported or very small with only 53 patients in the junior resident group.

### Independent risk factors for complications

We found that resident involvement was not a risk factor for either surgical site or systemic complications. For TKAs our results showed that higher age was associated with a decreased risk of surgical site complications, while a higher ASA score predicted an increased risk. For systemic complications, independent risk factors were male sex, DM, ASA III/IV, and higher age. Our results are partly in line with the results found in the literature (Haughom et al. 2014a). In the literature, increased operative time is found to be an independent risk factor for TKAs, however, this could not be confirmed in our study. Additionally, in the literature, higher age is indicated as a risk factor for complications (Pugely et al. 2013). This is confirmed only for systemic complications.

For THAs a longer operative time was associated with a higher risk of surgical site complications. For systemic complications, higher age, male sex, smoking, and a higher ASA score were found to be independent risk factors. Male sex as an independent risk factor has not been found or noted in previous literature. A risk factor that has been found in literature, but not in our study, was obesity (Haughom et al. 2014b). This may be explained by the fact that that specific study included BMI as categorical variable instead of continuous.

Despite the fact that the operative time in the resident group was longer and a longer operative time was associated with a higher risk of surgical site complications, no more surgical site complications were found. This is an important point for teaching hospitals. In our study, the resident was the first surgeon in only 29% of the TJA procedures. Given the fact that no more complications were found, it may be considered to have more TJA procedures performed by the resident, with due regard for the prolonged operative time. This may benefit the training of surgical skills during residency.

### Strengths and limitations

In our study each individual patient chart was reviewed, and data was crosschecked with the complication registries of the departments and with the data in the Dutch Arthroplasty Register. Furthermore, each surgical report was reviewed to ascertain the role of the resident during surgery. The study has a large sample size and a long follow-up. As a result, a reliable number of complications have been captured and reliable conclusions can be drawn concerning the complication risk and risk of early revisions. National revision rates are in the same ranges as the revision rates found in this study and the distribution of ASA score is more or less equal to the national distribution.

Limitations might be the retrospective design of the study. Further, there is probably a selection bias due to the fact that the orthopedic surgeon will operate on the more difficult patients. Also, patients might have expressed their preference or might have refused a resident as primary surgeon. This has not been recorded in the patient files. No information is available on minimal clinically significant differences regarding differences in complications. A non-significant difference might be experienced as a clinically relevant difference by patients. In future research, preoperative radiographs could be used to classify the surgical difficulty of the operation to clarify possible selection bias. In addition, other results could be studied, such as patient-reported outcome measures, to analyze the effects of resident involvement.

### Conclusion

Surgical site complications, revisions, and mortality were similar in TKAs or THAs performed by the resident as primary surgeon compared with surgeries performed by an orthopedic surgeon. This data can be used in teaching hospitals and may help to reassure patients.

## Supplementary Material

Supplemental Material

## Supplementary data

Tables 3–4 and 6–7 are available as supplementary data in the online version of this article, http://dx.doi.org/10.1080/17453674.2021.1979296

DB and NW had full access to all of the data in the study and take responsibility for the integrity of the data and the accuracy of the data analysis. All authors were involved with the concept and design, acquisition, analysis, and interpretation of data. Statistical analysis and drafting of the manuscript: DB and NW. Critical revision of the manuscript for important intellectual content: all authors. Supervision: RP, DK, DD

*Acta* thanks Aare Märtson and Petri Virolainen for help with peer review of this study.
