# Supplementary material for: Resident training does not influence the complication risk in total knee and hip arthroplasty
Source: Acta Orthop. 2021 Oct 4;92(6):689–94. doi: 10.1080/17453674.2021.1979296 (PMC8635675; doi:10.1080/17453674.2021.1979296)
Supplement: Supplemental Material [file IORT_A_1979296_SM5097.pdf]

Supplementary data

Table 3. Multivariate analyses for surgical site complications by total knee arthroplasty

Patient variables	B (SE) [a]	OR (CI) [b]
Resident as primary surgeon	0.13 (0.15)	1.1 (0.9–1.5)
Age (years)	–0.35 (0.01)	1.0 (1.0–1.0)
BMI	–0.02 (0.02)	1.0 (1.0–1.0)
Duration of surgery (minutes)	0.00 (0.00)	1.0 (1.0–1.0)
Female sex	–0.10 (0.15)	0.9 (0.7–1.2)
Diabetes mellitus (no = reference)	0.03 (0.19)	1.0 (0.7–1.5)
ASA score II (ASA I = reference)	0.40 (0.20)	1.5 (1.0–2.2)
ASA III/IV (ASA I = reference)	0.56 (0.27)	1.8 (1.0–3.0)
Smoking (no = reference)	0.12 (0.20)	1.1 (0.8–1.7)
Regional anesthesia (general = reference)	–0.21 (0.15)	0.8 (0.6–1.1)

[a] B (SE): unstandardized regression weight with standard error.
[b] OR (CI): odds ratio with 95% confidence interval.
R^2 = 0.03 (Nagelkerke). Model $\chi^2(10) = 31$, $p = 0.001$.

Table 4. Multivariate analyses for systemic complications by total knee arthroplasty

Patient variables	B (SE) [a]	OR (CI) [b]
Resident as primary surgeon	0.27 (0.16)	1.3 (1.0–1.8)
Age (years)	0.07 (0.01)	1.1 (1.1–1.1)
BMI	0.01 (0.02)	1.0 (1.0–1.1)
Duration of surgery (minutes)	0.00 (0.00)	1.0 (1.0–1.0)
Female sex	–0.32 (0.16)	0.7 (0.5–1.0)
Diabetes mellitus (no = reference)	0.41 (0.18)	1.5 (1.1–2.1)
ASA score II (ASA I = reference)	0.31 (0.24)	1.4 (0.9–2.2)
ASA score III/IV (ASA I = reference)	0.83 (0.28)	2.3 (1.3–4.0)
Smoking (no = reference)	0.07 (0.25)	1.1 (0.7–1.7)
Regional anesthesia (general = reference)	–0.19 (0.16)	0.8 (0.6–1.1)

[a] B (SE): unstandardized regression weight with standard error.
[b] OR (CI): odds ratio with 95% confidence interval.
R^2 = 0.09 (Nagelkerke). Model $\chi^2(10) = 102$, $p < 0.001$.

Table 6. Multivariate analyses for surgical site complications by total hip arthroplasty

Patient variables	B (SE) [a]	OR (CI) [b]
Resident as primary surgeon	–0.16 (0.16)	0.9 (0.6–1.2)
Age (year)	0.00 (0.01)	1.0 (1.0–1.0)
BMI	0.01 (0.02)	1.0 (1.0–1.0)
Duration of surgery (min)	0.01 (0.00)	1.0 (1.0–1.0)
Female sex	–0.03 (0.14)	1.0 (0.7–0.1)
Diabetes mellitus (no = reference)	0.29 (0.21)	1.3 (0.9–2.0)
ASA score II (ASA I = reference)	–0.04 (0.18)	1.0 (0.7–1.4)
ASA score III/IV (ASA I = reference)	0.47 (0.24)	1.6 (1.0–2.6)
Smoking (no = reference)	–0.11 (0.19)	0.9 (0.6–1.3)
Regional anesthesia (general = reference)	0.01 (0.16)	1.0 (0.7–1.4)
Diagnosis osteoarthritis (other = reference)	0.36 (0.21)	1.4 (0.9–2.2)

[a] B (SE): unstandardized regression weight with standard error.
[b] OR (CI): odds ratio with 95% confidence interval.
R^2 = 0.02 (Nagelkerke). Model $\chi^2(11) = 22$, $p = 0.03$.

Table 7. Multivariate analyses for systemic complications by total hip arthroplasty

Patient variables	B (SE) [a]	OR (CI) [b]
Resident as primary surgeon	0.09 (0.14)	1.1 (0.8–1.5)
Age (year)	0.05 (0.01)	1.1 (1.0–1.1)
BMI	–0.02 (0.02)	1.0 (1.0–1.0)
Duration of surgery (min)	0.00 (0.00)	1.0 (1.0–1.0)
Female sex	–0.35 (0.14)	0.7 (0.5–0.9)
Diabetes mellitus (no = reference)	0.05 (0.21)	1.1 (0.7–1.6)
ASA II score (ASA I = reference)	0.45 (0.22)	1.6 (1.0–2.4)
ASA score III/IV (ASA I = reference)	1.40 (0.26)	4.1 (2.5–6.7)
Smoking (no = reference)	0.41 (0.18)	1.5 (1.1–2.1)
Regional anesthesia (general = reference)	–0.11 (0.15)	0.9 (0.7–1.2)
Diagnosis osteoarthritis (other = reference)	0.16 (0.23)	1.2 (0.8–1.8)

[a] B (SE): unstandardized regression weight with standard error.
[b] OR (CI): odds ratio with 95% confidence interval.
R^2 = 0.11 (Nagelkerke). Model $\chi^2(11) = 153$, $p < 0.001$.
